# Identification of the Granule-Bound Starch Synthase (GBSS) Genes Involved in Amylose Biosynthesis in Tartary Buckwheat (*Fagopyrum tataricum* (L.) Gaertn.)

**DOI:** 10.3390/plants14020203

**Published:** 2025-01-13

**Authors:** Juan Huang, Fei Liu, Jieqiong Zhang, Bin Tang, Jiao Deng, Taoxiong Shi, Liwei Zhu, Hongyou Li, Qingfu Chen

**Affiliations:** 1 Research Center of Buckwheat Industry Technology, College of Life Science, Guizhou Normal University, Guiyang 550025, China; huang200669@163.com (J.H.); 18386012438@gznu.edu.cn (F.L.); 19010100309@gznu.edu.cn (B.T.); ddj613@163.com (J.D.); shitaoxiong@gznu.edu.cn (T.S.); 201505005@gznu.edu.cn (L.Z.); hongyouluod@163.com (H.L.); 2 Guizhou Provincial Agricultural Technology Extension Station, Guiyang 550001, China; gzsnjz@126.com

**Keywords:** Tartary buckwheat (*Fagopyrum tataricum* (L.) Gaertn.), granule-bound starch synthase (GBSS), amylose biosynthesis, gene expression pattern, cis acting elements (CAEs), transcription factors (TFs), transgenic

## Abstract

Tartary buckwheat is a nutrient-rich pseudo-cereal whose starch contents, including amylose and amylopectin contents, and their properties hold significant importance for enhancing yield and quality. The granule-bound starch synthase (GBSS) is a key enzyme responsible for the synthesis of amylose, directly determining the amylose content and amylose-to-amylopectin ratio in crops. Although one has already been cloned, the *GBSS* genes at the genome-wide level have not yet been fully assessed and thoroughly analyzed in Tartary buckwheat. This study comprehensively analyzed the *FtGBSSs* in Tartary buckwheat. Based on the genome data of Tartary buckwheat, five *FtGBSS* genes, namely *FtGBSS-1* to *FtGBSS-5*, were identified on three chromosomes, exhibiting about 1800 bp lengths in their CDSs and numerous exons and introns in gene structures. Amino acid analyses revealed high homology in ten GBSS proteins from Tartary buckwheat, rice, maize, and *Arabidopsis thaliana*, with a specific starch synthase catalytic domain and ten conserved motifs. The Tartary buckwheat GBSS proteins had a closer relationship with GBSS proteins from monocot based on evolutionary relationship analysis. Expression analyses suggested that the *FtGBSS* genes showed distinct tissue-specific expression patterns in Tartary buckwheat and rice-Tartary buckwheat. Among them, *FtGBSS-1*, *FtGBSS-2*, and *FtGBSS-4* were higher expressed in the root, stem, or flower, suggesting that they have a role in the amylose synthesis of these tissues. Notably, *FtGBSS-3* and *FtGBSS-5* were more highly expressed in seeds than in other tissues, suggesting that they have a pivotal role in amylose synthesis of the seeds of Tartary buckwheat. Furthermore, the cis acting elements in the promoters of *FtGBSSs* and their binding transcription factors (TFs) were investigated. A protein–protein interaction network was constructed and co-expression was analyzed based on the gene expression patterns of the *FtGBSSs*, and the identified TFs, belonging to bZIP, ERF, bHLH, and MADS-box TF families, were identified within this network, and their expression patterns were significantly correlated to the expression patterns of two seed-specific *FtGBSS* genes (*FtGBSS-3* and *FtGBSS-5*). Finally, *FtGBSS1-5* was successfully transformed into rice through transgenic manipulation, and the *FtGBSS1-5* overexpression lines showed an increase in amylose content accompanied by a reduction in amylopectin and total starch contents compared with WT. Overall, this research not only deepens our understanding of the molecular mechanisms of amylose synthesis in Tartary buckwheat, but also provides scientific insights for enhancing crop amylose content and quality through molecular breeding.

## 1. Introduction

Buckwheat, belonging to the Polygonaceae family and *Fagopyrum* genus, serves as a pseudo-cereal that is nutritious, healthy, and resilient to barren conditions, such cold and salt [1,2,3,4]. It plays a pivotal role in optimizing crop planting structures, improving soil fertility, and promoting poverty alleviation through agricultural development. Cultivated buckwheat is primarily classified into two categories: sweet or common buckwheat (*Fagopyrum esculentum*) and bitter or Tartary buckwheat (*Fagopyrum tataricum*) [5,6]. Tartary buckwheat, as a crop with both medicinal and edible properties, has been greatly favored by consumers because it is greatly beneficial to human health. The content of dietary fiber, resistant starch, and storage protein in seeds is significantly higher than that in major grain crops [1,7,8,9,10,11,12]. Furthermore, it is rich in flavonoids, such as rutin and quercetin, which can balance nutrition, boost immunity, and reduce hypertension, hyperglycemia, and hyperlipidemia, bringing multiple benefits to our human health [13,14]. Meanwhile, buckwheat grains and sprouts are a valuable gluten-free functional food source, rich in high-quality amino acids like methionine and leucine, with notable early-stage accumulation of glutamic acid and related amino acids [15].

Starch is the primary component of Tartary buckwheat seeds, which exhibit extensive application due to their potential and unique health benefits. The composition and structure of Tartary buckwheat starch have a significant impact on the quality of buckwheat-based foods [7,16]. The total starch content in Tartary buckwheat seeds ranges from 45% to 78%, with amylose and amylopectin as the main components, accounting for 10–28% and 23–60%, respectively [7,17]. The amylose content positively correlates with cooking loss, while the amylopectin content significantly affects the water absorption, viscosity, and hardness of noodles [18]. In terms of morphological structure, Tartary buckwheat starch granules exhibit irregular polygonal shapes with sharp edges, similar to but slightly larger than those of common buckwheat starch [19]. Their diameters are smaller than those of general crops but larger than those of rice (*Oryza sativa* L.) starch [19]. The crystal structure of Tartary buckwheat starch is of type A, a characteristic closely related to factors such as starch source, variety, amylose, and amylopectin content, as well as growth conditions [20]. Tartary buckwheat starch exhibits unique gelatinization properties, with higher viscosity and breakdown peak values compared with common buckwheat starch, and it requires less time and energy for gelatinization [21]. Notably, Tartary buckwheat is rich in resistant starch, with a content ranging from 45.1 to 105.2 mg/g [8]. This type of starch is not absorbed in the small intestine but has lower glycemic index and thus is helpful for preventing intestinal diseases and promoting probiotics [22]. Due to Tartary buckwheat’s lower glycemic index and higher resistant starch content, it can serve as a gluten-free alternative [16,23].

The starch synthesis pathway has been well studied in major crops such as rice, maize (*Zea mays* L.), and wheat (*Triticum aestivum* L.). Sucrose produced through photosynthesis is unloaded and distributed by GRAIN INCOMPLETE FILLING 1 (GIF1). After undergoing a series of reactions and transport processes, sucrose is converted into adenosine diphosphate glucose (ADPG) by the action of adenosine diphosphate glucose pyrophosphorylase (AGPase). Subsequently, ADPG is transported to the amyloplasts via Brittle 1 (Bt1) for starch synthesis. In cereal endosperm, amylose is synthesized by granule-bound starch synthase (GBSS), while the synthesis of amylopectin requires the coordinated cooperation of starch synthases (SSs), starch branching enzymes (SBEs), and starch debranching enzymes (DBEs) [24,25,26].

GBSS plays a crucial role in the synthesis of amylose by continuously adding glucose residues from ADPG to the non-reducing end of glucan through α-1,4-glycosidic bonds, effectively elongating the linear structure of glucan. The GBSS family includes two isoforms, GBSSI (or GBSS1) and GBSSII (or GBSS2). GBSSII functions in non-storage plant tissues, where transient starch accumulates, while GBSSI primarily plays a key role in storage tissues such as endosperm [26,27]. At the genetic level, the cDNA lengths of the GBSSI genes typically range from 0.6 to 2.4 kb, containing 6 to 13 exons, and encode proteins with a molecular weight of approximately 30 to 70 kD. GBSSII proteins have a larger molecular weight, ranging from 70 to 100 kD, and their amino acid sequence share high homology with GBSSI [27]. The cereal GBSS genes are also known as *waxy* (*wx*). Mutants of *waxy* in rice, maize, barley (*Hordeum vulgare* L.), and wheat show no significant change in total starch content in the endosperm, but a significant reduction or even complete absence of amylose [28,29,30]. In addition, the physicochemical properties of starch in the *waxy* mutant are also changed, resulting in alterations to the eating quality and cooking quality of grains [28,29,30]. Four waxy rice starches with different physicochemical properties are obtained by editing the *waxy* gene in rice using CRISPR/Cas9. The results show that the amylose content of these *waxy* mutants range from 0.26% to 1.78%, and the gel consistency, water solubility, number of short chains, degree of polymerization, and gelatinization temperature vary among different mutants [28]. Mutations in the *GBSSI* gene in maize result in high amylopectin content, leading to the formation of waxy corn, which has a texture widely favored [29]. Mutation in the GBSS gene from one waxy barley results in a 90% reduction in the catalytic activity of the HvGBSSIa enzyme, and shows deficient starch targeting mostly into unknown subcellular bodies of 0.5–3 mu m in size, which affects amylose synthesis, leading to the formation of high-amylopectin waxy barley [30].

Simultaneously, the process of amylose synthesis by GBSS is regulated by numerous transcription factors (TFs), including those belonging to the bZIP family, MYC-like, AP2/ERF, NAC, DOF, MADS, and NF-Y [31,32,33,34,35,36,37,38,39]. These TFs can directly and specifically bind to the specific cis-acting elements (CAEs) on the promoter of the GBSS gene, thereby modulating GBSS expression and subsequently influencing amylose synthesis. For instance, several TFs within the bZIP family in rice, including REB (also named as bZIP33), RISBZ1 (also named as bZIP58), and OsbZIP20, can specifically bind to the ACGT motif on the GBSS promoter, regulating GBSS expression and affecting the amylose content and starch composition in rice grains, which in turn influence rice taste [31,32,33]. Another example is two NAC family TFs in maize, ZmNAC128 and ZmNAC130, which can specifically bind to the ACGCAA element on the promoters of multiple target genes, including *BT2*, *Zpu1*, *GBSSI*, *Sh2*, *SSV*, *ISA2*, and *SSIIa*, thereby regulating their expression and impacting the starch and protein content in maize kernels [36].

Despite the deeply studied molecular mechanism underlying amylose synthesis in crops such as rice, maize, and wheat, research in this area for Tartary buckwheat remains less thorough. Wang et al. cloned the first *FtGBSS* gene in Tartary buckwheat, namely *FtGBSS1*. This gene consists of 14 exons and 13 introns, showing 63.3–75.1% homology with GBSS genes in dicotyledonous plants and 56.6–57.5% homology with those in monocotyledonous plants [40]. Li et al. investigated the expression pattern of the *FtGBSSI* gene, revealing a high correlation between the measured amylose content and *FtGBSSI* gene expression in mature Tartary buckwheat [41]. In recent years, some comparative transcriptomics were performed in the grain filling stage of Tartary buckwheat and, thus, five GBSS genes were identified from Tartary buckwheat and potential gene regulatory networks were proposed including the co-expression of *GBSS*, TFs, and brassinosteroids [42,43,44]. However, the *FtGBSS* genes have not been further characterized. Therefore, this study cloned the five *GBSS* genes based on the genomic data of Tartary buckwheat, and analyzed their gene structures, chromosomal locations, physicochemical properties of the encoded amino acids, multiple sequence alignments, and phylogenetic relationships using bioinformatics. Quantitative reverse transcription PCR (qRT-PCR) was employed to identify the tissue specificity of the five *FtGBSSs*. Additionally, the presence of the binding sites that directly bind to *GBSS* promoters reported in other crops, was analyzed in the promoters of the five *FtGBSSs*. The identified TFs with cis-acting elements (CAEs) on the FtGBSS promoters, their expression patterns, protein–protein interaction network, and correlations were also analyzed. Finally, a gene predominantly expressed in seeds, *FtGBSS-5,* was further studied, and its biological function in amylose synthesis was validated through transgenic rice. This study preliminarily elucidates the role of *GBSS* genes during Tartary buckwheat seed development and provides a theoretical basis for effectively regulating amylose synthesis in Tartary buckwheat, which is of great significance for improving Tartary buckwheat starch quality and properties.

## 2. Results

### 2.1. Gene Identification and Gene Structure Analysis of the FtGBSS Genes

**Figure 1:**
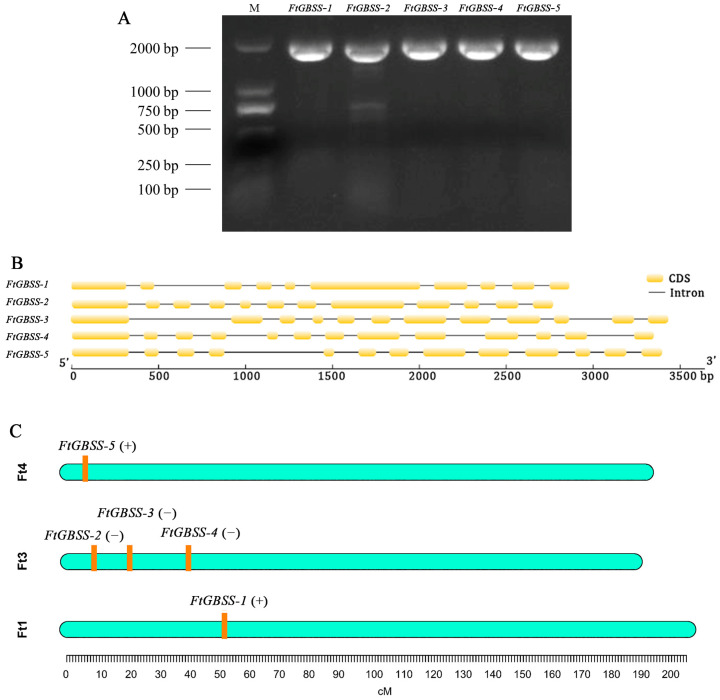
Gene cloning (**A**), gene structure (**B**) and chromosome localization (**C**) of *GBSS* genes in Tartary buckwheat.

All GBSS protein sequences were retrieved from the rice and *Arabidopsis thaliana* genomic databases. A local BLASTP (1 × 10^−5^) was performed using them as a query and using the Tartary buckwheat genome data as a database, resulting in five homologous sequences designated as *FtGBSS-1*, *FtGBSS-2*, *FtGBSS-3*, *FtGBSS-4*, and *FtGBSS-5* (Table 1). Gene-specific primers were designed to clone the five GBSS genes from Tartary buckwheat cDNA (Figure 1A). Their coding sequence (CDS) lengths are 1803, 1815, 1824, 1818, and 1815 bp, and encoding 600, 604, 604, 605, and 607 amino acids, respectively. The predicted protein molecular weights are 65.493, 66.692, 66.398, 66.604, and 66.467 kDa, respectively. The predicted isoelectric points (pI) are 7.27, 6.53, 5.92, 7.95, and 6.07, respectively. The predicted aliphatic indices are 89.37, 82.76, 86.04, 88.74, and 85.02, respectively. The predicted protein hydrophobicity values (GRAVY) are −0.077, −0.236, −0.126, −0.137, and −0.153, respectively. The values of all five GRAVY are negative, indicating that the GBSS proteins of Tartary buckwheat are hydrophilic. Among them, FtGBSS-2 has the strongest hydrophilicity with a hydrophobicity value of −0.236, while FtGBSS-1 has the weakest hydrophilicity with a hydrophobicity value of −0.077 (Table 1).

Further analysis of the gene structures of the five *FtGBSS* genes revealed that they possess a relatively high number of introns (Figure 1B). Specifically, *FtGBSS-2* and *FtGBSS-3* both contain 12 exons and 11 introns, while *FtGBSS-4* and *FtGBSS-5* both contain 13 exons and 12 introns. *FtGBSS-1*, on the other hand, contains 10 exons and 9 introns. Based on the chromosomal locations of the five *FtGBSS* genes, *FtGBSS-1* is located on chromosome 1 (Ft1) in a forward orientation, whereas *FtGBSS-2*, *FtGBSS-3*, and *FtGBSS-4* are all arranged in the reverse orientation on chromosome 3 (Ft3). Additionally, *FtGBSS-5* is found to be oriented in the forward direction on chromosome 5 (Ft5) (Figure 1C, Table 1).

### 2.2. Amino Acids Analysis and Phylogenetic Tree Construction of the FtGBSS Proteins

**Figure 2:**
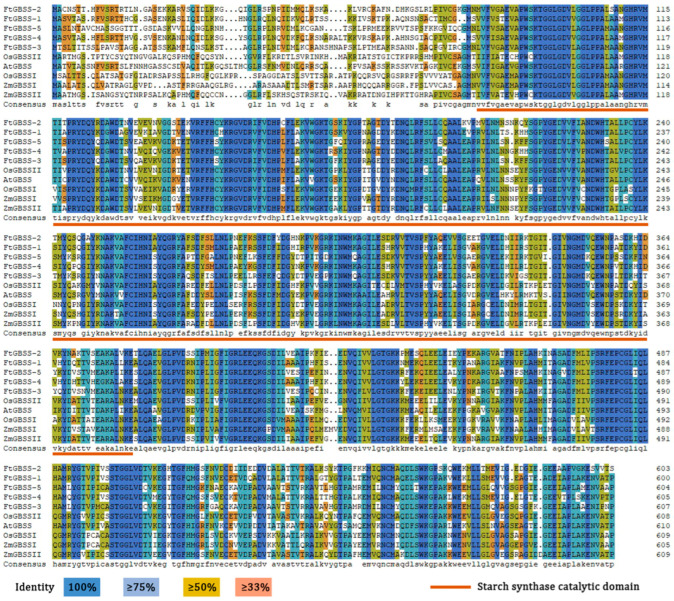
Multiple amino acid sequences alignment of GBSS proteins.

Amino acid multiple sequence alignment was performed between the five GBSS protein sequences from Tartary buckwheat and five GBSS protein sequences from rice, maize, and *Arabidopsis thaliana*, revealing a high homology at their amino acid level (Figure 2). Furthermore, all these GBSS proteins contain a specific starch synthase catalytic domain (IPR013534), which represents the catalytic domain of starch synthases that utilize ADP-glucose as the glucose donor.

**Figure 3:**
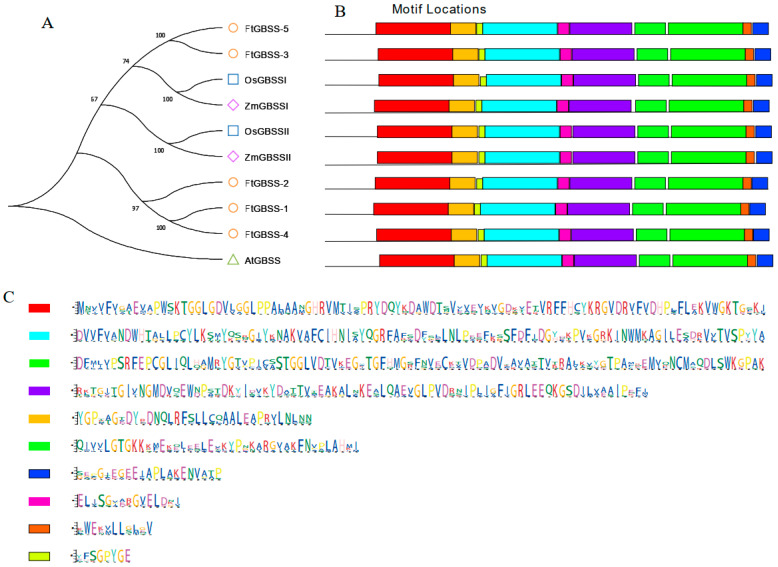
Phylogenetic tree (**A**) and the conserved motifs (**B**,**C**) of the GBSS proteins. Different colors in the rectangles of (**B**,**C**) represent different motifs.

Subsequently, to gain insights into the evolutionary relationship of the GBSS proteins, phylogenetic analysis was conducted on these ten GBSS proteins. The results show that the GBSS protein from the dicotyledonous plant *Arabidopsis thaliana* clusters separately, while the other nine GBSS proteins form a single cluster (Figure 3A). FtGBSS-3 and FtGBSS-5 share the same branch with OsGBSS1 and ZmGBSS1, whereas FtGBSS-1, FtGBSS-2, and FtGBSS-4 form a distinct branch, suggesting that they exhibit closer relationships and may have similar functions. Meanwhile, the MEME tool was employed, and ten conserved motifs alongside their respective motif logos were identified (Figure 3B,C). Notably, while the N-terminal region of the GBSS proteins exhibits considerable divergence, suggesting potential functional specialization, the C-terminal region remains strikingly conserved, implying a crucial role in maintaining structural integrity or essential enzymatic functions. All the ten conserved motifs are presented across the GBSS protein family members, which suggests that these motifs are fundamental to the function of GBSS enzymes, highlighting the high degree of conservation within this family in plants.

### 2.3. Expression Analysis of Five FtGBSS Genes in Tartary Buckwheat

**Figure 4:**
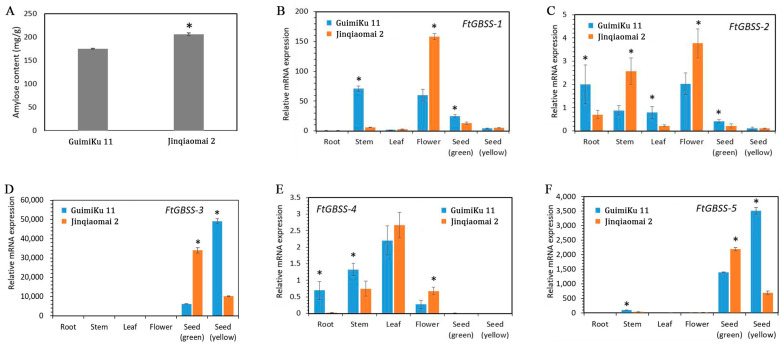
Expression patterns of five *FtGBSS* genes in different tissues of Tartary buckwheat. (**A**) The amylose content between Jinqiaomai 2 and GuimiKu 11. (**B**–**F**) Relative mRNA expression level in different tissues between two varieties. The error bars represent the standard deviations of the replicates. Asterisks show the statistical significance evaluated by Student’s *t* test. Seed (green) is in short of the seeds in the final filling stage (with green shells). Seed (yellow) is in short of the seeds in maturity stage (with yellow shells).

Jinqiaomai 2 and GuimiKu 11, two representatives of Tartary buckwheat were selected to evaluate the expression of the five FtGBSS genes. It was found that the amylose content in Jinqiaomai 2 is significantly higher than that in GuimiKu 11 (Figure 4A). Therefore, we analyzed the expression patterns of five FtGBSS genes in different tissues, including roots, stems, leaves, flowers, and seeds in the final filling stage (with green shells) and seeds in the maturity stage (with yellow shells) from these two varieties. The results indicate that they exhibit distinct expression patterns in different tissues (Figure 4B–F). In the different tissues of Guimiku 11, *FtGBSS-1* exhibits the higher expression levels in stems and flowers; *FtGBSS-2*, and *FtGBSS-4* are higher expressed in roots, stems, leaves, and flowers; *FtGBSS-3* and *FtGBSS-5* are preferentially expressed in seeds. In the different tissues of Jinqiaomai 2, *FtGBSS-1* is preferentially expressed in flowers; *FtGBSS-2* is higher expressed in roots, stems, and flowers; *FtGBSS-4* is higher expressed in stems, leaves, and flowers; *FtGBSS-3* and *FtGBSS-5* are preferentially expressed in seeds. These findings suggest that *FtGBSS-3* and *FtGBSS-5* primarily contribute to the synthesis of amylose in Tartary buckwheat seeds, regardless of the varieties.

Notably, the expression of the five FtGBSS genes are different in the same tissues between two varieties (Figure 4B–F). Specifically, significant differential expressions between the two varieties are observed for *FtGBSS-1* in stems, flowers, and seeds in the final filling stage; for *FtGBSS-2* in roots, stems, leaves, flowers, and seeds in the final filling stage; for *FtGBSS-3* in the seeds in the final filling stage and in the maturity stage; for *FtGBSS-4* in roots, stems, and flowers; and for *FtGBSS-5* in stems and seeds in the final filling stage, and seeds in the maturity stage.

### 2.4. Cis Acting Elements (CAEs) Analyses on the Promoters of FtGBSSs and Identification of the Potential Binding Transcription Factors (TFs)

**Figure 5:**
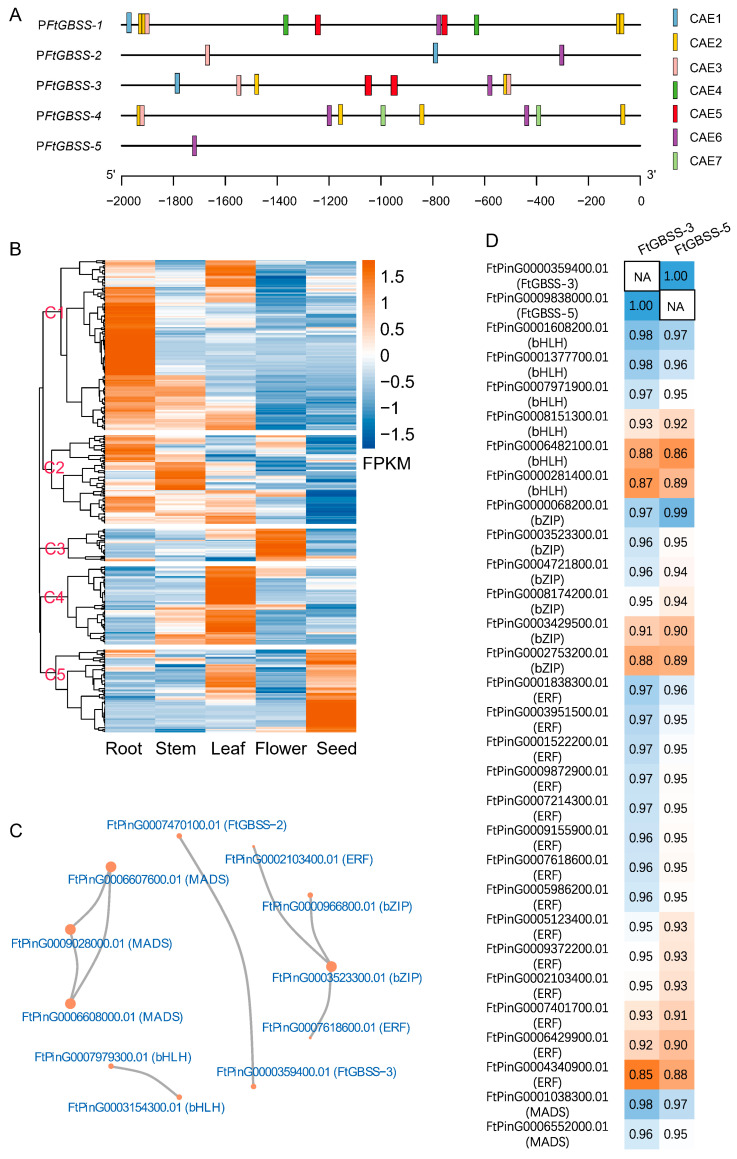
Cis acting elements (CAEs) analyses on the promoters of FtGBSSs and identification of the potential binding transcription factors (TFs). (**A**) Localization of the CAEs identified on the promoters of five *FtGBSSs*. CAE1, prolamin-box (P-box); CAE2, ACGT motif; CAE3, ACGTGG; CAE4, GCN4 motif; CAE5, CArG-box; CAE6, GCCAAC; CAE7, [AG][CT]CGAC. (**B**) Hierarchical clustering of the expression patterns of five TF family genes in different tissues. (**C**) The protein–protein interaction (PPI) network of TFs and *FtGBSSs* that were dominant expressed in seeds. (**D**) The TFs whose expression patterns exhibit significant correlation (*p* < 0.05) with the expression patterns of two seed-specific *FtGBSS* genes (*FtGBSS-3* and *FtGBSS-5*).

Based on previous reports, several TF family members, including those belonging to the bZIP, MYC-like bHLH, ERF, NAC, DOF, and MADS families, have been shown to bind to the promoters of GBSS genes, which directly regulate their expression and consequently influence the amylopectin content [31,32,33,34,35,36,37,38]. Thereby, we obtained the cis-acting elements (CAEs) that specifically bind with these transcription factors. Subsequently, we extracted the 2000 bp sequences on the upstream region of the translation initiation site (ATG) for the five FtGBSS genes and employed local Python analysis to screen for the presence of potential CAEs that bind with these TFs. Our finding reveals the existence of 32 CAEs for five TF families (DOF, bZIP, bHLH, MADS, and ERF) within the promoters of the five GBSS genes (Figure 5A). These includes one DOF CAE (CAE1, prolamin-box, P-box), two bZIP CAEs (CAE2, ACGT motif; CAE4, GCN4 motif), one bHLH CAE (CAE3), one MADS CAE (CAE5, CArG-box), and two ERF CAEs (CAE6 and CAE7). Furthermore, our analysis reveals that the upstream regions of the five FtGBSS genes contain varying numbers of these cis-acting elements, with counts of 11, 3, 8, 9, and 1, for FtGBSS-1, FtGBSS-2, FtGBSS-3, FtGBSS-4, and FtGBSS-5, respectively.

We subsequently retrieved the complete sequences of five TF families (DOF, bZIP, bHLH, MADS, and ERF) from the model crop rice. Employing local BLASTP against the predicted protein sequences of the Tartary buckwheat genome, we identified 30, 100, 152, 63, and 117 members of the DOF, bZIP, bHLH, MADS, and ERF TF families, respectively, at the genome-wide level. To gain insights into their expression patterns, we analyzed transcriptome data from five tissues of Tartary buckwheat, including roots, stems, leaves, flowers, and seeds reported in previous studies. Hierarchical clustering of the expression profiles classifies these genes into five distinct clusters (C1–C5, Figure 5B). Cluster C1 includes 152 genes that exhibit high expression levels in roots and low expression in flowers and seeds. Cluster C2 comprises 79 genes with high expression in roots and stems but low expression in seeds. Cluster C3 includes 29 genes that are predominantly expressed in flowers. Cluster C4 has 70 genes that are highly expressed in leaves but show lower expression in seeds and roots. Lastly, cluster C5 contains 74 genes that are specifically upregulated in seeds.

Starch content in grains is a pivotal trait, comprising roughly 70% of the total grain weight. Consequently, our attention is focused on the TFs belonging to cluster C5 that are predominantly expressed in the seeds of Tartary buckwheat. To understand their interactions, we constructed a protein–protein interaction (PPI) network, incorporating these 74 TFs belonging to cluster C5 alongside the *FtGBSS* genes. The result reveals intricate interactions among these proteins (Figure 5C). Notably, direct interactions exist between *FtGBSS-2* and *FtGBSS-5*, indicating their potential collaborative role in starch biosynthesis. Furthermore, three MADS family TFs (FtPinG0006607600.01, FtPinG0009028000.01, and FtPinG0006608000.01) are shown to engage in direct PPIs, highlighting their possible coordinated regulation. Similarly, two bHLH family TFs (FtPinG0007979300.01 and FtPinG0003154300.01) form direct connections, suggesting they might function together in modulating starch-related processes. Interestingly, a bZIP family TF (FtPinG0003523300.01) acts as a pivot, connecting with two ERF TFs (FtPinG0002103400.01 and FtPinG0007618600.01) and another bZIP member (FtPinG0000966800.01). These networks suggest the complex regulatory mechanism underpinning starch synthesis in Tartary buckwheat seeds.

It is worthwhile mentioning that the expression levels of *FtGBSS-3* and *FtGBSS-5* in seeds vastly exceed those in roots, stems, leaves, and flowers, by several thousand to ten thousand-fold (Figure 4). This suggests their seed-specificity and essential function in starch synthesis of Tartary buckwheat seed. Notably, the expression patterns of these two genes exhibit a remarkable similarity, with a correlation coefficient of 1.00, highlighting their tightly coordinated function (Figure 5D). To gain insights into their regulatory mechanisms, we analyzed the correlation between the expression patterns of 74 seed-specific TFs and two GBSS genes, resulting in 28 TFs that displayed significant correlations with two GBSS genes (Figure 5D). Among these TFs, six bHLH family members (FtPinG0001608200.01, FtPinG0001377700.01, FtPinG0007971900.01, FtPinG0008151300.01, FtPinG0006482100.01, FtPinG0000281400.01) showed significant correlations with a Person R ranging from 0.86 to 0.98; six bZIP family members (FtPinG0000068200.01, FtPinG0003523300.01, FtPinG0004721800.01, FtPinG0008174200.01, FtPinG0003429500.01, FtPinG0002753200.01) showed significant correlations with a Person R ranging from 0.88 to 0.99; fourteen ERF family members (FtPinG0001838300.01, FtPinG0003951500.01, FtPinG0001522200.01, FtPinG0009872900.01, FtPinG0007214300.01, FtPinG0009155900.01, FtPinG0007618600.01, FtPinG0005986200.01, FtPinG0005123400.01, FtPinG0009372200.01, FtPinG0002103400.01, FtPinG0007401700.01, FtPinG0006429900.01, FtPinG0004340900.01) showed significant correlations with a Person R ranging from 0.85 to 0.97; two MADS family members (FtPinG0001038300.01, FtPinG0006552000.01) showed significant correlations with a Person R ranging from 0.95 to 0.98. These findings suggest that these TFs may play important regulatory roles in concert with the GBSS genes to govern starch synthesis in Tartary buckwheat seeds.

### 2.5. Heterologous Transformation of FtGBSS-5 Increases the Amylose Content in Rice

**Figure 6:**
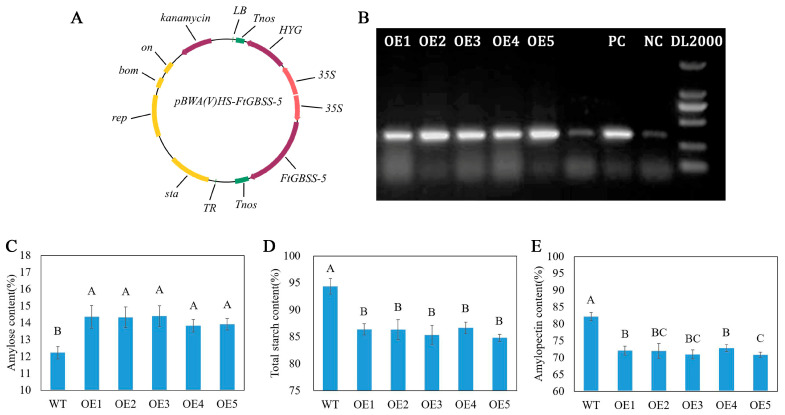
Heterologous transformation of *FtGBSS-5* in rice. (**A**) Vector construction of *FtGBSS-5*. (**B**) PCR identification of positive rice lines of *FtGBSS-5*. OE1–OE6, over-expression lines, PC, positive control (plasmid presented in **A**), NC, negative control (wild type rice). (**C**) Amylose content in the *FtGBSS-5* OE lines. (**D**) Amylopectin content in the *FtGBSS-5* OE lines. (**E**) Total starch content in the *FtGBSS-5* OE lines. Different capital letters on the bars represent significant differences (*p* < 0.05), while the same letters represent no significant differences among different lines.

The *FtGBSS-5* gene, with its exceptionally high expression in seeds, suggests a specific role in seed development. It is thus hypothesized that this gene is primarily responsible for the synthesis of amylose in Tartary buckwheat seeds. To experimentally confirm its function, a plant overexpression vector, designated as pBWA(V)HS-*FtGBSS-5* (Figure 6A), was constructed and employed for the heterologous transformation of rice. As a result, five transgenic rice lines (OE1–OE5) were obtained successfully (Figure 6B). A comparative analysis of starch content was conducted between these transgenic lines and wild-type rice. The results reveal that all five transgenic rice lines exhibit a significant increase in amylose content, compared to wild-type rice (Figure 6C). Conversely, the amylopectin content is significantly lower in the transgenic lines than that in the wild-type (Figure 6D). Furthermore, the total starch content in the transgenic rice is also found to be significantly reduced compared to the wild-type (Figure 6E). These findings conclusively demonstrate that the introduced *FtGBSS-5* gene effectively promotes amylose synthesis while inhibiting amylopectin synthesis in rice, ultimately enhancing the amylose content in the transgenic rice grains.

## 3. Discussion

The GBSS genes are involved in the synthesis of amylose, which is crucial for the grain yield and quality, especially the taste, of food crops. However, there is only one report on the identification and functional analysis of GBSS in Tartary buckwheat [40]. In this study, five GBSS genes (*FtGBSS-1* to *FtGBSS-5*) were identified. These five genes exhibit similarities in terms of CDS length, gene structure, predicted protein molecular weight, pI, aliphatic index, and GRAVY values, falling within the range reported for GBSSs in other species such as rice and wheat [26]. At the same time, they also demonstrate diversity, indicating differences in their physicochemical properties and potential functions. It is worth mentioning that rice, wheat, and barley have two copies of the GBSS gene each, maize has three, while Tartary buckwheat has up to five copies [26]. This suggests that there is significant variation in the copy number of GBSS genes among different species, which may be closely related to their starch synthesis capacity, structural characteristics of starch granules, and the need to adapt to different growth environments and ecological niches. Studies have shown that adverse environmental conditions, such as low temperature, nitrogen deficiency, and drought can all adversely affect photosynthesis, leading to inadequate grain filling and ultimately reducing crop yields [45,46,47]. Tartary buckwheat originates from the Himalayan regions of southwestern China [48], where the areas are characterized by high altitudes, low temperatures, and infertile soil. In order to adapt to these harsh growth conditions, Tartary buckwheat may have undergone certain adaptive changes during evolution, one of which is an increase in the copy number of the GBSS gene. This change aids Tartary buckwheat in completing the grain-filling process under extreme conditions, ensuring that the grains possess reproductive capability, thereby allowing the species to continue. Through multiple amino acid sequence alignments, it is shown that the five FtGBSS proteins in Tartary buckwheat exhibited high homology at the amino acid level with the GBSS proteins of rice, maize, and *Arabidopsis thaliana*, and all of them contain a starch synthase catalytic domain (IPR013534), which is a unique catalytic domain specific to glycogen (or starch) synthases that use ADP-glucose as a donor.

Though the conserved motifs within the GBSS proteins exhibit no obvious difference, their phylogenetic trees showed distinct subgroups. FtGBSS-3 and FtGBSS-5 are closer to OsGBSSI and ZmGBSSI, which are specifically expressed in the grains of rice and maize, and mutants of them directly affect the *waxy* gene of rice and maize [49,50]. Based on qRT-PCR results, the expression levels of *FtGBSS-3* and *FtGBSS-5* in seeds are thousands, or even tens of thousands of times higher than in other tissues, suggesting that they are seed-specific genes. Their expression patterns are similar to that of *OsGBSSI* and *ZmGBSSI*, indicating that they may also be key genes determining the synthesis of amylose and potentially affecting the *waxy* genes of Tartary buckwheat grains. However, further work is needed to confirm this hypothesis, such as analyzing whether there are sequence differences in these two genes among Tartary buckwheat varieties with different amylose contents. In contrast, FtGBSS-1, FtGBSS-2, and FtGBSS-4 clustered together on the evolutionary tree and formed a separate branch. These genes exhibit higher expression levels in non-seed tissues than in seeds based on qRT-PCR analysis, which suggests these genes may share similar functions and are involved in the amylose biosynthesis in non-seed tissues of Tartary buckwheat.

For CAEs analysis in the promoters of five *FtGBSS* genes, each gene’s promoter contained 1–11 CAEs belonging to the DOF, bZIP, bHLH, MADS, or ERF families, indicating that these TFs may regulate *FtGBSS* genes. Among these, 28 TFs predominantly expressed in seeds are significantly correlated with *FtGBSS-3* and *FtGBSS-5*. These include six bHLH family members, six bZIP family members, fourteen ERF family members, and two MADS family members. There are also instances where these TF families regulate amylose synthesis in other crops by binding to the promoters of GBSS genes. For example, a MYC protein contains a putative basic helix-loop-helix-ZIP DNA-binding domain, designated OsBP-5, can bind to the CAACGTG motif within the rice *wx* gene when OsEBP-89, a member of the EREBP family, is present, suggesting the OsBP-5 and OsEBP-89 proteins act synergistically to regulate the transcription of the rice *wx* gene [34]. OsbZIP58, a bZIP transcription factor, acts as a key transcriptional regulator controlling starch synthesis in rice endosperm by binding directly to the promoters of six starch-synthesizing genes (*OsAGPL3*, *wx*, *OsSSIIa*, *SBE1*, *OsBEIIb*, and *ISA2*) to regulate their expression [32]. A AP2/ERF family TF, SERF1, is a direct upstream regulator of *GBSSI* and represents a negative regulator of grain filling by timing the expression of RPBF in rice [35]. OsMADS14, interacting with nuclear factor NF-YB1, can directly bind to the CArG-box in the promoters of *OsAGPL2* and *Waxy* to promote their transcription [38]. These suggest that these TFs may be potential regulators that bind to the promoter of *FtGBSS-3* or *FtGBSS-5* to govern starch synthesis in Tartary buckwheat seeds. Subsequent experiments, such as yeast one hybrid, are needed to characterize the binding of *FtGBSS* genes and these TFs.

Hetero transformation of *FtGBSS-5* to rice successfully obtained five transgenic rice lines. Compared with the wild type rice, the *FtGBSS-5* transgenic lines show a significant increase in amylose content in seeds and a corresponding decrease in amylopectin and total starch content. This indicates that *FtGBSS-5* encods a key enzyme involved in amylose synthesis in Tartary buckwheat seeds. Among other crops such as rice, maize, and barley, mutations of GBSS showed a significant reduction or absence of amylose, resulting in the waxy grains, and thus has a great impact on the taste, nutritional value, eating quality, and cooking quality of grains [27,28,29,30]. Since *FtGBSS-5* is a key gene involved in the amylose synthesis of Tartary buckwheat seeds, we can subsequently screen Tartary buckwheat germplasm with different amylose contents and analyze the SNP/InDel variations in the sequence of *FtGBSS-5* among them to explore the correlation between sequence variations and amylose content and to identify superior alleles of *FtGBSS-5*. This will lay the foundation for molecular breeding of Tartary buckwheat with high amylose content.

## 4. Materials and Methods

### 4.1. Plant Planting and Sampling

Two Tartary buckwheat varieties, Jinqiaomai 2 and Guimiku 11, were selected and cultivated in the start of March and harvested at the end of June of 2021 at the Anshun experimental field (26°17′ N, 106°18′ E, 1200 m altitude), which is affiliated with the Buckwheat Industry Technology Research Center at Guizhou Normal University and belongs to a typical plateau-type humid subtropical monsoon climate. For these months, the average high and low temperatures were 21.75 °C and 14.50 °C, respectively, and the total precipitation was 127.4 mm (derived from the website: https://www.tianqi24.com/anshun/history202103.html, available at 4 January 2025). The soil type of the farmland is yellow clay soil. Based on local experience, the Tartary buckwheat was managed using conventional field management practices.

During the flowering phase, various tissue samples, including roots, stems, leaves, and flowers, were harvested from both Jinqiaomai 2 and Guimiku 11. Additionally, seed samples were collected at the late-grain filling stages and initial maturity stages.

As for rice, Zhonghua 11 was chosen for the transgenetic experiment. Seedlings were germinated and grown on a nutrient medium initially in a controlled environment chamber, maintained at 25 °C with a 16 h photoperiod (light intensity ranging from 7000 to 9000 Lx), an 8 h dark period, and a humidity level of 80%. Subsequently, these seedlings were transplanted into pots and placed in the growth chamber of the Buckwheat Industry Technology Research Center for further cultivation, adhering to standard cultivation practices. New rice leaves during rice booting stage were sampled for the following experiment.

To ensure reproducibility, each Tartary buckwheat or rice material was sampled in triplicate as three biological replicates. The samples were immediately frozen in liquid nitrogen and subsequently stored in a −80 °C freezer for future experiments.

### 4.2. Identificaioni and Bioinformatics Analyses of FtGBSSs

A total of five GBSS proteins were retrieved from the NCBI database (https://www.ncbi.nlm.nih.gov/ accessed on 26 September 2024). These included one GBSS protein in *Arabidopsis thaliana* (AtGBSS, accession number AAN31102), two GBSS proteins in rice (OsGBSSI, accession number AAF72561; OsGBSSII, accession number BAC21549), and two GBSS proteins in maize (ZmGBSSI, accession number CAA27574; ZmGBSSII, accession number NP_001334833). To identify the GBSS members in Tartary buckwheat, a local BLASTP (E-value < 1 ×10^−5^) was performed using the identified GBSS proteins as queries against the predicted protein sequences of the Tartary buckwheat genome data as the database [3]. The obtained sequences were compared with the lengths of known GBSS proteins, the specific starch synthase catalytic domain (IPR013534), evolutionary relationships, and amino acid motifs, ultimately identifying five potential GBSS homologs in Tartary buckwheat. Gene annotation information and chromosomal positions were extracted from the genome gff file. Subsequently, the protein molecular weight and theoretical pI of Tartary buckwheat GBSS proteins were analyzed using the ExPASy online Protparam tool (https://web.expasy.org/protparam/ accessed on 26 September 2024). The gene structures of five *FtGBSS* genes were analyzed with the Gene Structure Display Server (GSDS 2.0) online software (http://gsds.gao-lab.org/index.php/ accessed on 26 September 2024), and chromosomal localization was visualized using the LinkageMapView package in R language (version 4.4.0).

A multiple amino acid sequence alignment of ten GBSS proteins from Tartary buckwheat, rice, maize, and *Arabidopsis thaliana* was carried out employing DNAMAN software (version 6.0, Lynnon Corp., Quebec, QC, Canada). The protein domains of five FtGBSSs were predicted by the InterPro website (https://www.ebi.ac.uk/interpro/ accessed on 26 September 2024). A phylogenetic tree was constructed in MEGA software (version X), using the neighbor-joining method with 5000 bootstrap and the pairwise deletion option [51]. The motifs on the GBSS proteins and their corresponding sequence logos were predicted using the MEME Suite (https://meme-suite.org/meme/tools/meme accessed on 26 September 2024).

### 4.3. RNA Extractio, Cloning and qRT-PCR Analyses of FtGBSSs

The total RNA was extracted from Tartary buckwheat samples using the RNAprep Pure Plant Plus Kit (DP441, TianGen Biotech Co., Ltd., Beijing, China) following the manufacturer’s protocol. The integrity of the RNA was assessed by agarose gel electrophoresis. The concentration and purity of the RNA samples were determined using a microspectrophotometer. Subsequently, first-strand complementary DNA (cDNA) was synthesized from 1 µg of the total RNA using the PrimeScript™ II 1st Strand cDNA Synthesis Kit (6210A, Takara Biomedical Technology Co., Ltd., Beijing, China).

Gene-specific primers for cloning and the qRT-PCR of five *GBSS* genes were designed using the Premier Primer 6 software (Table 2). Using the mixed cDNA from different tissues of Guimiku 11 as a template, PCR amplification was performed using the Ex Taq Hot Start enzyme (RR006A, Takara Biomedical Technology Co., Ltd., Beijing, China). The reaction system and program were conducted according to the manufacturer’s instructions. After electrophoresis on a 1% agarose gel, the target band was excised and purified using a DNA recovery kit (DP209, TianGen Biotech Co., Ltd., Beijing, China). The purified target DNA was then ligated to the pGM-T vector (VT202, TianGen Biotech Co., Ltd., Beijing, China) at 16 °C overnight. DH5α competent cells were transformed with the ligation product, subjected to heat shock and cultivation, and then plated on LB medium containing ampicillin. The plates were incubated at 37 °C overnight. The next day, the monoclones were picked for cultivation, and PCR identification of the bacterial suspension was performed, then the positive monoclones were sequenced (Sangon Biotech Co., Ltd., Shanghai, China).

Gene-specific primers for qRT-PCR were designed using the Primer3Plus web tool (https://www.primer3plus.com// accessed on 26 September 2024) (Table 2). A pair of universal primer for Tartary buckwheat Actin genes was used as the inner reference gene [52]. qRT-PCR was conducted using the TB Green^®^ Premix Ex Taq™ II kit (RR820A, Takara Biomedical Technology Co., Ltd., Beijing, China) on an ABI 7500 Fast Real-Time PCR system (Thermofisher, Waltham, MA, USA). Each reaction was performed in triplicate for technical reproducibility. The cycling conditions were set according to the manufacturer’s instruction. Gene expression levels were quantified using the 2^−ΔΔCT^ method. Statistical analyses were conducted using SPSS Statistics software (Version 26, IBM, New York, NY, USA) with Student’s *t*-test to determine the statistical significance. The data were visualized using Office Excel software (version 2019, Microsoft, Redmond, DC, USA).

### 4.4. Promoter Analysis and Identification of Interacting TFs

We extracted the 2000 bp sequences upstream of the ATG start codon of the five GBSS genes from the Tartary buckwheat genome data, considering them as promoter regions. Using local Python scripts, we searched for multiple known TF binding sites, namely CEAs that have been reported to interact with GBSS promoters. Specifically, the bZIP family binds to the ACGT motif (GCCACGT[AC]AG) [31,33] and the GCN4 motif (TGA[GC]TCA) [32] in GBSS promoters; the bHLH family recognizes ACGTGG [34]; the ERF family binds to GCCAAC [35] and [AG][CT]CGAC [34]; the NAC family recognizes ACGCAA [36]; the DOF family binds to the prolamin-box (P-box, with the sequence TGTAAG) [37]; and the MADS family interacts with the CArG-box (CC[AT](6100)GG) [37]. We utilized Python scripts to analyze the presence and locations of these CAEs within the promoters of the five GBSS genes and employed TBTools for visualization [53].

The full sequences of the six transcription factor families mentioned above in rice were retrieved from the Plant Transcription Factor Database (https://planttfdb.gao-lab.org/ accessed on 26 September 2024). BLASTP was then performed against the predicted protein sequences of the Tartary buckwheat genome [3] to predict the members of these six transcription factor families in Tartary buckwheat. Transcriptome data from the Tartary buckwheat genome [3] was obtained and used to analyze the expression patterns of FtGBSS genes and their associated TFs across different tissues. The hierarchical cluster was performed using the “pheatmap” package in R, and correlation analysis and plotting were performed using the “corrplot” package in R [54]. STRING (https://cn.string-db.org/ accessed on 26 September 2024) was utilized to conduct a protein–protein interaction network analysis of related proteins, and the results were visualized using the “igraph” package in R.

### 4.5. Heterologous Transformation of FtGBSS-5 into Rice and Starch Content Determination

The full-length CDS of the *FtGBSS-5* gene was ligated into the pBWA(V)HS vector using the In-Fusion^®^ HD Cloning Kit (639648, Takara Biomedical Technology Co., Ltd., Beijing, China), following the manufacturer’s instructions. The primers used for vector construction, designated as pBWA(V)HS-FtGBSS-5, were designed on an online platform (https://www.takarabio.com/learning-centers/cloning/primer-design-and-other-tools accessed on 26 September 2024) (Table 2). *Bsa* I and *Eco31* I were employed as the double restriction sites for the ligation.

Subsequently, the rice variety Zhonghua 11 was selected as the transgenic plant receptor, and the rice transformation experiment was conducted by BIORUN BIOSCIENCES CO., Ltd., Wuhan, China. The transgenic seeds were screened on 1/2 MS medium containing hygromycin resistance, and the positive seedlings were transferred to nutrient soil for normal growth. The positive transgenic rice lines were identified through PCR using the vector-specific 35S as the forward primer and a gene-specific reverse primer (Table 2). The pBWA(V)HS-*FtGBSS-5* construct served as the positive control, while wild-type rice was used as the negative control.

The determination of amylose and amylopectin content followed the same method as we previously used, and the total starch content was the sum of the amylose and amylopectin content [17].

## 5. Conclusions

This study performed a comprehensive analysis of the FtGBSS gene family in Tartary buckwheat, identifying five FtGBSS genes and deeply investigating their structures, expression patterns, and relationship with starch synthesis, significantly advancing our understanding of the amylose synthesis mechanism in Tartary buckwheat. Through comparative analysis, it was found that FtGBSSs showed significant tissue-specific expression patterns, among which *FtGBSS-1*, *FtGBSS-2*, and *FtGBSS-4* were higher expressed in the root, stem, or flower, whereas *FtGBSS-3* and *FtGBSS-5* were more highly expressed in seeds than in other tissues. A complex regulatory network involving multiple TF families was investigated. The crucial role of *FtGBSS-5* in seed amylose synthesis was further confirmed. This study not only deepens our understanding of the molecular mechanisms underlying amylose synthesis in Tartary buckwheat but also provides valuable insights for enhancing crop amylose content and quality through molecular breeding.

## Figures and Tables

**Table 1:** Information of the *FtGBSS* genes in Tartary buckwheat.

Gene ID	New Gene Name	Length of CDS/bp	Length of Amino Acids	Molecular Weight/kDa	Theoretical pI	Liphatic Index	GRAVY	Chromosome (Orientation)
FtPinG0000380300.01	*FtGBSS-1*	1803	600	65.493	7.27	89.37	−0.077	Ft1:50850592-50853642 (+)
FtPinG0007470100.01	*FtGBSS-2*	1815	604	66.692	6.53	82.76	−0.236	Ft3:11190277-11193653 (−)
FtPinG0000359400.01	*FtGBSS* *-* *3*	1824	604	66.398	5.92	86.04	−0.126	Ft3:17156120-17159629 (−)
FtPinG0005565800.01	*FtGBSS-4*	1818	605	66.604	7.95	88.74	−0.137	Ft3:38354014-38358126 (−)
FtPinG0009838000.01	*FtGBSS-5*	1815	607	66.467	6.07	85.02	−0.153	Ft4:6323422-6327187 (+)

The plus (+) and minus (−) signs in chromosome orientation represent that the gene is oriented on the chromosome in the forward and reverse directions, respectively.

**Table 2:** Primers used in this study.

Name	Forward Primer	Reverse Primer	Product Length (bp)	Purpose
FtGBSS-1c	ATGGCTAGCGTAACTGCATC	TCTTAGGGAGTTGGAACATTTT	1820	Gene cloning
FtGBSS-2c	ATGGCTAGTGTGACAGCGTCTC	TTAGGGGGTTGCGACATTTTCC	1803	Gene cloning
FtGBSS-3c	ATGACGTCACTCACAATCACA	AGCCTCCTGATTATGGGTT	1834	Gene cloning
FtGBSS-4c	ATGGCTTGCAACAGTACAAC	CTGGAAATAACACCTTAAGGAC	1829	Gene cloning
FtGBSS-5c	ATGGCGTCTCTGAATACCG	GCATATATCAAGGAGCAGCAAC	1815	Gene cloning
FtGBSS-1q	GAGGTTGGCTTACCGGTTGA	AATTCCTCTGGCCTTGTCCG	210	qRT-PCR
FtGBSS-2q	CGCTGAGTTCAGGAGTTCGT	GCTTCCTTCAGGAGTGCCTT	312	qRT-PCR
FtGBSS-3q	GAGGTGGGATTGCCAGTTGA	CCGGTTCCCAGAACGAGAAA	143	qRT-PCR
FtGBSS-4q	AGCCGTGTGGTCTCATTCAG	TTTCGACGGTCCCTTCCAAG	272	qRT-PCR
FtGBSS-5q	CCAGCCATGCGAGAGATGAT	GGGCAATTTCTTCCCCCTCA	139	qRT-PCR
FtActUniv	GAGTTATGAGCTTCCTGATG	CCGCCACTCAACACAATGTT	192	qRT-PCR
pBWA(V)HS- *FtGBSS-5*	cagtggtctcacaacATGGCGTCTCTGAATACCGC	cagtggtctcatacaTCAAGGAGCAGCAACATTTT	1815	Vector construction
pBI-*G5*-iden	GACGCACAATCCCACTATCC (35S)	TTTTTGAAGCTGGGACTCGT	215	Identification of positive *FtGBSS-5* transgenetic *rice* lines

Note: Lowercase letters represent the restriction enzyme cutting sites.

## Data Availability

The genomic data and transcriptome data of Tartary buckwheat are available from http://www.mbkbase.org/Pinku1/, accessed on 25 September 2024.
